# Palynological evidence for pre-agricultural reindeer grazing and the later settlement history of the Lycksele region, northern Sweden

**DOI:** 10.1007/s12520-021-01275-7

**Published:** 2021-02-13

**Authors:** Ilse M. Kamerling, J. Edward Schofield, Kevin J. Edwards

**Affiliations:** 1 Department of Geology and Geophysics, School of Geosciences, University of Aberdeen, Meston Walk, Aberdeen, AB24 3UE UK; 2 Department of Geography and Environment, School of Geosciences, University of Aberdeen, Elphinstone Road, Aberdeen, AB24 3UF UK; 3 Department of Archaeology, School of Geosciences, University of Aberdeen, Elphinstone Road, Aberdeen, AB24 3UF UK; 4 McDonald Institute for Archaeological Research and Scott Polar Research Institute, University of Cambridge, Cambridge, UK

**Keywords:** Forest Sami, Reindeer herding, Nordic farmers, Colonization, Grazing, Hay making, Fire clearance, Swidden, Boreal forest, Pollen analysis, Coprophilous fungal spores

## Abstract

Analyses of high-resolution pollen data, coprophilous fungal spores, microscopic charcoal and sedimentology, combined with radiocarbon dating, allow the assessment of the impact of Sami and Nordic land use in the region surrounding the winter market town of Lycksele in northern Sweden. Such winter markets were established by the Crown during the seventeenth century AD to control the semi-nomadic movements of the Sami who traded here with Finnish settlers and were also taxed and educated. Little is known about Sami and Nordic co-existence beyond these market places, mainly due to a lack of archaeological evidence relating to Sami activity. Vegetation and land-use changes in the region between ~ AD 250 and 1825 reveal no signal for pre-seventeenth century agricultural activity, but the coprophilous fungal spore records suggest the increased regional presence of grazing herbivores (possibly reindeer) between ~ AD 800 and 1100. Sami activity in the parish of Lycksele has been suggested by rich metal finds dated to ~ AD 1000–1350 and they may have been attracted by an abundance of reindeer.

## Introduction

**Fig. 1:**
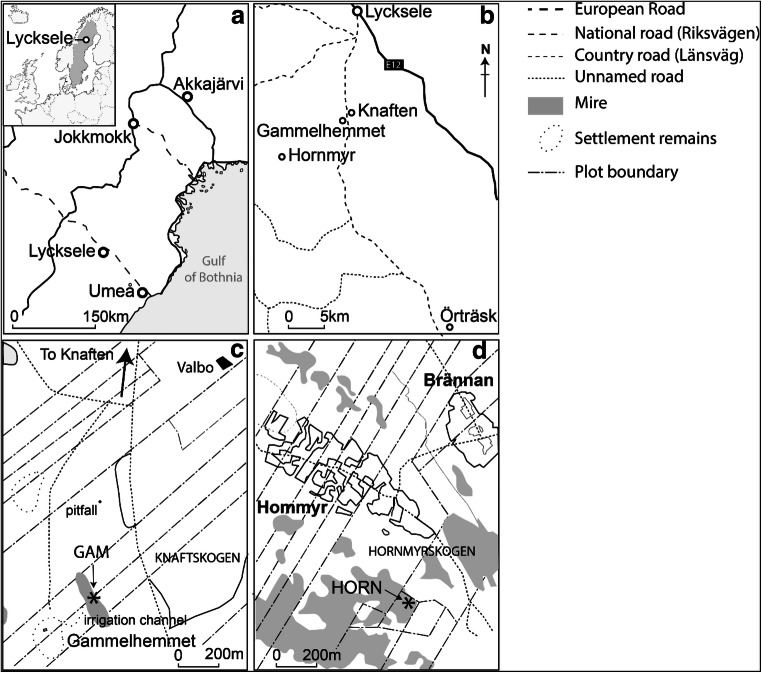
**a** Locations of winter market towns Lycksele, Umeå and Jokkmokk in northern Sweden; **b** Locations of the two pollen-analysed sites, Gammelhemmet and Hornmyr, and their relationship to Lycksele, Knaften and Örträsk; **c** Detailed map of the sampling location at Gammelhemmet (GAM); **d** Detailed map of the sampling location at Hornmyr (HORN)

This paper reports the findings of palynological investigations at the Nordic farming settlements of Gammelhemmet-i-Knaften and Hornmyr within the catchment of the market town of Lycksele, in Västerbotten province, northern Sweden (Fig. 1). Such markets formed the main centres of cultural interaction between the Sami and Nordic agricultural settlers, and offer an opportunity to study the activities of both cultural groups. As far as the authors are aware, no previous palynological research has been undertaken in this region. The aims of our investigation were as follows: (i) to use pollen analysis and associated proxies to confirm ideas regarding the introduction of agriculture, and the Nordic settlement history of the area; (ii) to consider whether a signal for Sami activity may be evident in the palynological record.

**Fig. 2:**
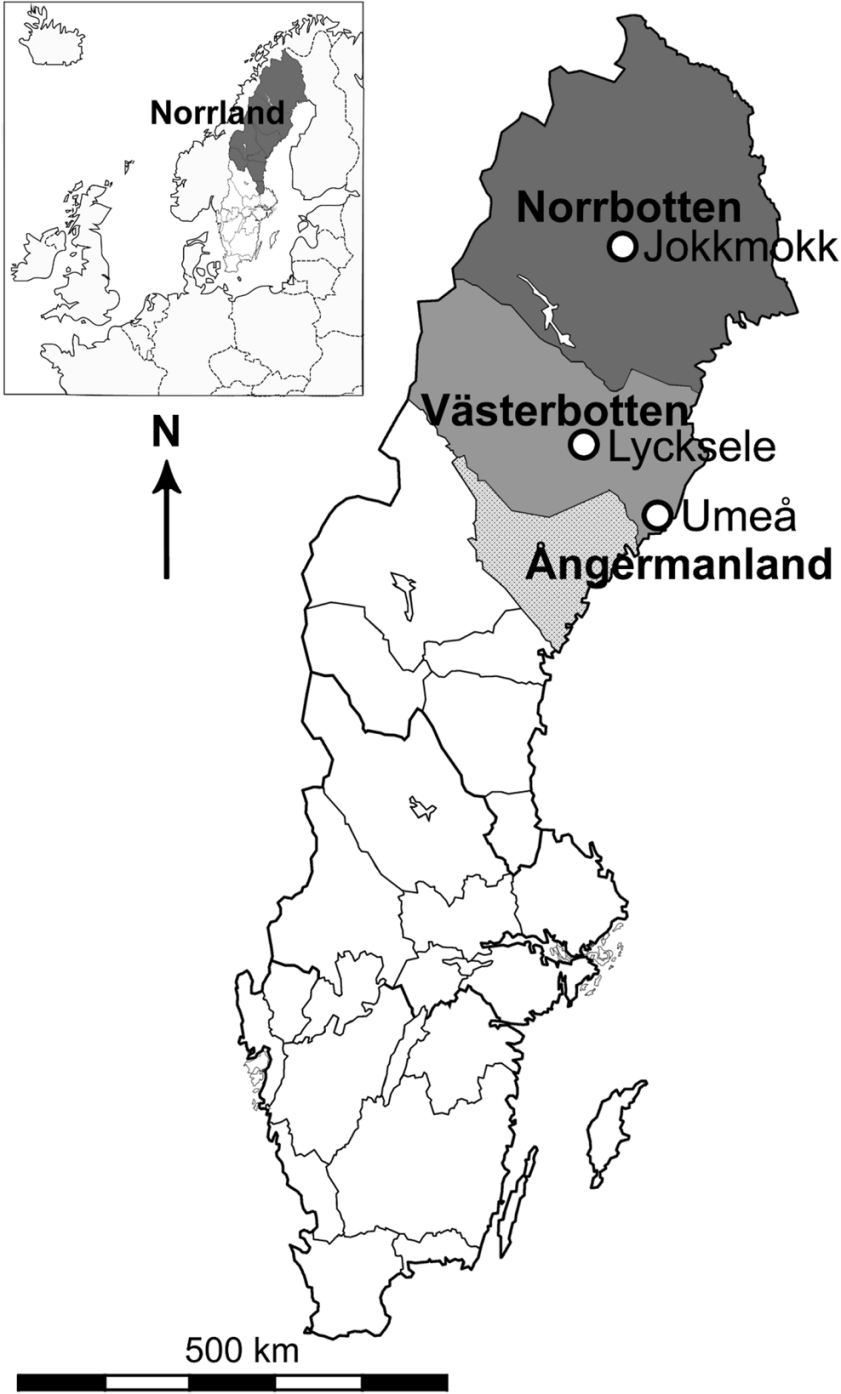
The location of the provinces of Norrbotten, Västerbotten and Ångermanland, which collectively constitute Norrland (indicated in dark grey on the inset map), within Sweden. The locations of market towns (Lycksele, Umeå and Jokkmokk) are indicated

Västerbotten is one of three provinces in Norrland (Fig. 2). The latter encompasses more than 50% of Sweden’s land area, but compared to southern Sweden (e.g. Gaillard and Berglund 1988; Lagerås 1996; Lindbladh 1999; Segerström and Emanuelsson 2002; Berglund et al. 2008), it is severely underrepresented in terms of land-use reconstructions. Based on the assumption that Sami hunter-gatherer activity formed the main subsistence strategy into historical times (SCB 1955; Segerström et al. 1994; Palm 2000; Lundmark 2007), human impact in Norrland has often been considered insignificant. Palaeoecological studies in northern Sweden have mainly focused on questions surrounding climate change, natural succession and the establishment of specific taxa (e.g. *Picea*) following deglaciation and disturbance by fire (e.g. Bradshaw 1993; Barnekow and Sandgren 2001; Giesecke and Bennett 2004; Hörnberg et al. 2004; Barnekow et al. 2008; Giesecke et al. 2008). At present, investigations of post-Stone Age human impacts on inland forest areas have been few (Van der Linden et al. 2008; Hörnberg et al. 2015), particularly where Swedish forest Sami reindeer herders are concerned (Aronsson 1991, 1994). Yet, palynological studies have long been applied globally to reconstruct low-impact human activity (Dimbleby 1985; Behre 1988; Edwards and MacDonald 1991; Bennett et al. 1992; Gardner 2002). This includes studies relating to the development of sedentary agriculture and Sami hunter-gatherer and reindeer (*Rangifer tarandus*) herding activity in inland Finland, Estonia and northern Sweden (Hicks 1988; Carpelan and Hicks 1995; Niinemets and Saarse 2009; Tallavaara et al. 2010; Kamerling et al. 2017; Hörnberg et al. 2018).

Despite being spread across Sapmi (the Sami homeland that geographically and politically covers northern Norway, Sweden, Finland and the Russian Kola Peninsula), much still remains unknown about the cultural development of the Sami due to the limited availability of historical and more diagnostic archaeological evidence. Evidence of a Sami presence in the form of hearths, cooking pits and hunting pits are abundant in the archaeological record of northern Sweden (Liedgren et al. 2007; Karlsson 2008; Lars et al. 2016), and radiocarbon dating of bone and charcoal fragments can provide information on the occupation history of such sites. They are not always complemented by other artefacts that would shed more light on the ways in which the site was used because Sami reindeer herding sites are highly transient and artefacts relating to reindeer domestication (e.g. harnesses) are largely organic and do not preserve well in the acidic podzols of northern Sweden (Aronsson 1991; Kibblewhite et al. 2015). At Pasvik in Arctic Norway, bones and other artefacts at hearth row sites have been linked to trade and metal processing, suggesting that Sami societies and economies were more complex than often supposed (Hedman et al. 2015). Archaeological finds may also not be ideally located in the vicinity of deposits suitable for palaeoenvironmental reconstruction, which, depending on basin size, may otherwise shed light on local land use, as well as more geographically extended human activity and/or climate change.

Boreal forest (taiga) vegetation can be significantly altered by human activity over prolonged intervals compared to its pristine natural state (Josefsson et al. 2009, 2010). The impact of such activities, including those of indigenous peoples, can be visible for centuries as the recovery of vegetation may take considerably longer than the duration of the activities that caused them (Josefsson et al. 2010; Freschet et al. 2014; Walker and Wardle 2014). Such a recovery typically follows a secondary successional trend from Poaceae-domination to ericaceous heaths and *Betula*, through to *Pinus* and finally *Picea* forests (Bradshaw and Zackrisson 1990; Jonsson and Esseen 1998; Freschet et al. 2014). Many artefacts and land-use patterns created by the Sami and/or sedentary agriculturalists have been destroyed by extensive logging practices throughout northern Sweden since the beginning of the AD 1800s (Östlund and Bergman 2006). Research into the past activity of Nordic agriculturalists and Sami has therefore focused either on high altitude alpine forests in the west (Salmonsson 2003; Karlsson et al. 2007, 2009; Staland et al. 2011; Östlund et al. 2015) or on coastal areas in the east (Engelmark 1976; Hörnberg et al. 2014). The wealth of peatlands in the interior of Norrland provides a vast and under-utilised palaeoecological archive of potential human- and climate-induced vegetation changes, and these are used here to examine historical activities in a region of cultural convergence between reindeer herders and farmers. This study uses coprophilous fungal spores—a valuable indicator of animal presence that was hitherto often-overlooked in this geographical setting—alongside pollen analysis and related proxies to provide new insight into the past inter-relationships between people, animals and the landscape.

## Background and context

### Forest Sami and their signal in the palaeoecological record

Reindeer have formed an important part of Arctic and sub-Arctic cultures and economies since the Palaeolithic (Sturdy 1975; Aikio 1989; Müller-Wille et al. 2006). Sami reindeer herding is one of five Eurasian types (Aronsson 1991) and is characterised by reindeer milking, dog-controlled pasturing, and the use of reindeer for the transport of goods/people and as decoys during hunting (Heinrich 2006; Müller-Wille et al. 2006).

Two sub-types of Sami reindeer herding are practised: the mountain and the forest type (Aronsson 1991; Niklasson et al. 1994; Norstedt and Östlund 2016). Mountain Sami migrate with their herds between summer mountain pastures and forest areas in the winter. Semi-nomadic forest Sami migrated strictly within the boreal forest (Aronsson 1991; Hedman 2003; Norstedt and Östlund 2016) and operated mainly in coastal areas, but were active as far south and inland as the interior of Ångermanland (Westerdahl 1986). Reindeer herding can be intensive or extensive, depending on the level of herd control (Aronsson 1991). Intensive herding was most suitable for the forest Sami and focused on managing small numbers of tame reindeer, with controlled calving and consumption of reindeer milk (Müller-Wille et al. 2006). The use of milking pens necessitated regular migrations to reduce the risk of calf diphtheria contracted from muddy soils, and of foot and mouth disease (Östlund et al. 2003). Pens were often abandoned for several years (Aronsson 1991) and patterns of use and abandonment can be visible in the palaeoecological record (Kamerling et al. 2017). Semi-nomadism was required to meet the subsistence needs of reindeer throughout the year (Heinrich 2006). The variety of soils in the boreal forests of northern Sweden provide a mosaic of different vegetation types that can be accessed through regular short migrations as the seasons change (Renbeteslära 1982). Together with hunting and fishing, semi-nomadic reindeer herding was the dominant type of land use in northern Sweden until the beginning of the eighteenth century (Aronsson 1994), though palaeoecological evidence of cereal cultivation in interior northern Sweden has been attributed to the Sami from as early as AD 800 (Bergman and Hörnberg 2015). These authors further argue that in many ways, the Sami use of plants for food was not merely ‘gathering’ but was rather a form of cultivation where yields were optimised and persistence of the plant communities of interest was safeguarded through the application of traditional knowledge.

Sami society was organised according to the *siida* village system comprised of multiple families or kin groups (Müller-Wille et al. 2006). The *siida* communities migrated between 3 and 7 semi-permanent settlement locations within their assigned areas from spring to autumn to meet the needs of their herd and themselves. In addition, forest Sami subsistence also relied on hunting and fishing, so pastures would be located near suitable fishing waters (Laestadius 1977; Graan 1983; Rheen 1983; Niklasson et al. 1994), where semi-permanent dwellings were set up (Aronsson 1991; Niklasson et al. 1994). During winter, the entire *siida* community travelled to the winter village where they met with (fur) traders, tax collectors and Nordic church ministers (Müller-Wille et al. 2006).

Non-agricultural human impacts within the boreal forest are usually visible in pollen records as an increase in apophyte abundance (Räsänen et al. 2007). An increase in a combination of Poaceae and certain herbaceous taxa (e.g. *Epilobium*-type, *Solidago*-type, *Ranunculus*, *Urtica*, Chenopodiaceae and Caryophyllaceae) have commonly been recorded at Sámi settlement sites (Aronsson 1991; Hicks 1993; Aronsson 1994). The clearance of trees and shrubs for (temporary) dwellings, and to create gathering spaces for reindeer, would have led to openings in the forest canopy, thus encouraging the growth of light-demanding herbaceous taxa, notably Poaceae, *Melampyrum pratense*, *M. sylvaticum* and *Solidago vigaurea* (Aronsson 1991). Trampling of the ground by reindeer over multiple weeks resulted in soil erosion and mixing. This attracted taxa that thrive on minerogenic soils affected by disturbance (e.g. *Epilobium angustifolium* and *Rumex acetosella*). Increased levels of sunlight reaching the forest floor combined with the destruction of the natural vegetation cause soil nitrification (Sjörs 1971). When allied with the addition of reindeer dung and the disposal of waste from the dwelling area, this would fertilise the soil, favouring nutrient-requiring vegetation such as *Galeopsis* (hemp-nettles) and *Urtica* (nettles).

Forest grazing generally reduces the availability of soil nutrients and can create a habitat for resistant plant genera such as *Juniperus*, *Melampyrum* and Ranunculaceae (Van der Linden et al. 2008). However, grazing has no noticeable effect on vegetation in the pasture grounds outside of the main reindeer gathering areas because reindeer are extensive grazers (Hicks 1985; Aronsson 1991).

### Nordic colonization and its signal in the palaeoecological record

Palaeoecological studies undertaken along the current Bothnian coastline in the provinces of Ångermanland and Västerbotten (Fig. 2) suggest that Nordic farmers arriving from the south settled in these areas from around AD 500 (Engelmark 1976; Wallin 1996), though the timing of the onset and the spread of cultivation is still under debate; a review of palynological evidence has not indicated a south to north spread (Josefsson et al. 2014) and permanent cereal cultivation already took place inland from ~ AD 480 (Josefsson et al. 2017). In any case, subsistence methods would have focused on livestock rearing supplemented by hunting, gathering and the low-intensity cultivation of *Hordeum* (Engelmark 1976; Wallin 1996; Pedersen and Widgren 2011). At this time, coastal areas were already occupied by Sami (Von Düben 1873; Broadbent 2004) who were considered largely unaffected by Nordic colonization up until ~ AD 1000 when the Swedish State started to impose taxes (Myrdal 2011). Settler populations grew and extended into inland forest areas from around AD 900, with colonization beginning in earnest from ~ AD 1100 supported by livestock rearing through transhumance (Engelmark 1976; Myrdal 2011; Pedersen and Widgren 2011). From around AD 1200, Sami who refused to convert to Christianity were prohibited from occupying Nordic-owned lands (Broadbent 2010). A further and greater wave of colonial expansion into the central and northern Swedish boreal forests occurred during the sixteenth and seventeenth centuries, executed by Finnish settlers (Abramsson et al. 2006; Wedin 2007). Finland had been under Swedish rule from the beginning of the thirteenth century and colonization of unoccupied lands in interior northern Sweden by *Finnskogar* or ‘Forest Finns’ was encouraged by the Swedish Crown, who relied on these settlers to defend such lands during times of conflict (Abramsson et al. 2006; Gadd 2011). The Finns were particularly effective at bringing areas under cultivation through the application of traditional slash-and-burn methods of cultivation (Pentikäinen 1995; Wedin 2007; Ekengren 2013). Around this time, King Karl IX sought to convert the Sami to Christianity through the establishment of designated churches and winter market places such as Lycksele, Jokkmokk and Umeå (Figure 1). Contact with the Sami was maintained here for the purposes of taxation and trade, the resolution of disputes, and the education of both Sami and settlers.

Much like the non-agricultural impact of hunter-gathering Sami, the impact of small-scale agriculture on the vegetation may be difficult to distinguish in the palynological record, but becomes stronger as the level of activity intensifies. Forest clearance for pasture and cultivation of cereals and other crops may be evident as a decrease in *Pinus* and *Picea* and increased levels of *Alnus* and *Betula*, combined with the introduction of Cerealia pollen, e.g. *Secale cereale* with the later appearance of *Hordeum*-type and in some cases *Triticum*-type, as well as the pollen of other crops such as *Cannabis*/*Humulus* (Engelmark 1976; Aronsson 1991; Wallin 1996). Alongside this, we may expect to find pollen of weeds that respond positively to an opening up of the canopy and/or weeds of cultivation: Poaceae, *Artemisia vulgaris*, *Rumex acetosa*/*acetosella*, *Plantago lanceolata*, *P*. *major*/*media*, *Galium*-type, Caryophyllaceae, Lactuceae, Chenopodiaceae, Asteraceae undiff., *Ranunculus*, *Epilobium*-type, *Filipendula*, *Salix* and *Juniperus*. Animal husbandry may be indicated by the presence of the grazing indicators Poaceae and *Juniperus* (Engelmark 1976; Van de Veen and Van Wieren 1980; Buttenschøn and Buttenschøn 1985). Many of these taxa are the same as those that are indicative of Sami hunter-gatherer and reindeer herding impacts on the environment. As such, it may be difficult to separate the two where both cultural groups may have been active in the same locations at the same time.

### Regional setting and cultural history

The parish of Lycksele is situated in the main boreal forest zone of Sweden (Sjörs 1963). In its northern section, ~ 35 km north of the town of Lycksele, around 53% of the forested area is occupied by pine, 1% by spruce, 21% by mixed coniferous forest and 25% by coniferous-deciduous forest. Ground floras are dominated by *Empetrum nigrum*, *Calluna vulgaris*, *Vaccinium* spp. and *Cladonia* spp. (Östlund et al. 1997). This composition is partly the result of extensive commercial logging activity and strict forestry regulations imposed over the past 150 years (Axelsson and Östlund 2001). Intensive forest management has converted natural communities into high-yielding, even-aged, forest stands that were clear-cut between 1970 and 1990 (ibid.). Quaternary sediments in the region are dominated by peat deposits and glacial till, underlain at Hornmyr by Svecofennian granites, and at Gammelhemmet by granitoids and metavolcanics (SGU 2007). Northwest-southeast orientated drumlins and moraines are common across the region.

Lycksele was established by agricultural settlers in AD 1607 at a traditional Sami meeting place (Abramsson et al. 2006). It sits within what was known as the Ume Sami district (*Ume lappmark*, later renamed *Lycksele lappmark*), which consisted of 37 Sami territories (Norstedt et al. 2014). The vast majority of these territories (28) belonged to forest Sami, a further eight belonged to mountain Sami, and the final territory that directly surrounded Lycksele belonged to the Church. Evidence of reindeer herding and Sami occupation at the *Gammplatsen* of Lycksele (the location of the church and market place, also known as an *Öhn*, meaning ‘old site’) is scarce (Abramsson et al. 2006; Rydström 2006). In the wider parish, evidence for Sami presence is limited to rich metal finds dated to ~ AD 1000–1350 (Zachrisson 1984). Finnish settlement in this region started with the establishment of Örträsk in AD 1678, ~ 40 km southeast of Knaften (Fig. 1) (Abramsson et al. 2006).

### Sites

#### Gammelhemmet-i-Knaften

Gammelhemmet is situated 15 km to the south of Lycksele (Fig. 1) within the Lycksele *prästbord* territory (Norstedt et al. 2014). It forms the oldest part of the village of Knaften, which was established in AD 1701 (Abramsson et al. 2006). The fields surrounding the old farm house, featuring several clearance cairns, are thought to have been used for hay making and perhaps cultivation, although poor soils and crop failure due to frost meant that stock breeding, hunting and fishing were required to supplement swidden (slash-and-burn) cultivation (Egerbladh 1963; Bergman and Ramqvist 2017; Skogsmuseet 2017). Gammelhemmet was abandoned after ~ 150 years because of periods of severe frost, and a new settlement was established where central Knaften is currently situated. Evidence of Sami utilisation of the area has been inferred from the presence of hunting pits several hundred metres northeast of the village and a *renvall* (reindeer herding pen) of unknown age and hunting pit systems within a radius of several kilometres of the site (Khorasani et al. 2015). The mire at Gammelhemmet is dominated by saplings of *Betula* spp. and *Salix*, with a field layer of *Sphagnum*, *Eriophorum vaginatum* and *Potentilla*. The surrounding woodland is dominated by *Betula*, *Picea* and *Pinus*.

#### Hornmyr

The village of Hornmyr is in the Lycksele *prästbord* and is located 25 km southwest of Lycksele (Fig. 1). It was established in AD 1766 (Abramsson et al. 2006) by Olof Andersson, a settler from Örträsk (Carrion 2017). As at Gammelhemmet, conditions at Hornmyr were seemingly suboptimal for settlement, with sandy and stony soils unfavourable for cultivation. Encouragement to settle here in the form of tax exemptions that exceeded the usual 15 years was therefore offered by the Swedish government. The vegetation on the mire consists mainly of *Picea*, scattered *Pinus*, *Betula* spp. (including *B. nana*), Poaceae, *Polytrichum*, *Eriophorum vaginatum* and *Sphagnum*. *Salix* grows in local drainage ditches.

## Methods

### Sample collection

Peat cores were collected from small to medium-sized mires in order to optimise the chances of detecting a palynological signal for settlement. In boreal forests, the dispersal of pollen types indicative of human impact through cultivation and hunter-gathering—mainly herbaceous taxa indicative of an opening up of the landscape—is generally limited to a radius of 20-30 m (Vuorela 1973; Hicks 1993), which is broadly equivalent to the theoretical pollen source areas defined for small basins of < 0.5 ha (Jacobson and Bradshaw 1981). Sugita (1998) has suggested that when attempting to detect local changes in landscape openness, basins with a surface area of 1–20 ha are suitable, within which those < 2 ha most ideally suited.

At Gammelhemmet, a core (coded GAM) was collected with an 8-cm diameter half metre Russian borer (Jowsey, 1966) from a mire approximately 200 m northeast of the main concentration of farm buildings (64° 25.95’ N, 18° 36.89’ E; Fig. 2). The site is adjacent to a pit excavated for entomological samples (Khorasani et al. 2015). The mire is small (~ 60 x 250 m; ~ 1.5 ha) and is situated at ~ 283 m asl. The sequence encompasses the interval 13-64 cm below the modern ground surface; the topmost 13 cm of the deposit was too friable for collection and the basal unit was impenetrable with the Russian corer. The peat sequence at Hornmyr (coded HORN) was collected with the same Russian borer at a location ~ 25 m to the west of a drainage ditch and an intensively mown area (64° 23.63' N, 18° 24.61' E; Fig. 2). This section of the mire has a surface area of ~ 190 x 280 m (5.3 ha) and is connected to a larger deposit (1875 x 440 m; ~ 82.5 ha).

### Sedimentary characteristics

Core stratigraphies were described using the Troels-Smith (1955) scheme. In order to detect small changes in the minerogenic content of the (highly organic) peat, loss-on-ignition (LOI) was performed by Thermogravimetric Analysis (TGA), which measures weight loss through combustion in a controlled environment to determine the percentage of inorganic matter by weight (Ball 1964; Beaudoin 2003). Analyses were conducted using a Leco Corporation TGA-601 in the Sediment Analysis Laboratory at the Vrije Universiteit in Amsterdam. Contiguous 1-cm-thick samples were dried in an oven (80 °C overnight), ground to a powder, heated to 105 °C to expel H_2_O, weighed, and combusted at 550 °C until weight loss had ceased (usually ~ 3 h).

### Palynology

Contiguous 1-cm-thick samples of ~ 1 cm^3^ were measured by volumetric displacement (Mooney and Tinner 2011). *Lycopodium* tablets (Stockmarr 1971) were added to allow the determination of palynomorph concentrations and influx. Pollen sample preparation followed conventional methods (Moore et al. 1991; Chambers et al. 2011) and included treatment with 10% NaOH and acetolysis. Samples were mounted unstained in silicon oil (12,500 cSt viscosity).

A counting sum of ≥ 500 total land pollen (TLP) was attained using a Nikon E400 binocular light microscope at × 400 magnification. Obligate aquatic taxa, spores and inferred exotic (long-distance derived) pollen types were excluded from the pollen sum. Slides were counted along evenly spaced transects across a half or a full slide to circumvent problems arising from any uneven distribution of palynomorphs. Pollen and spores were identified using the key in Moore et al. (1991) and the reference collection held in the Department of Geography and Environment, University of Aberdeen. Nomenclature largely follows Bennett (2020a). *Betula* pollen grains were systematically measured, with those < 20 μm classified as *B. nana* (dwarf birch), and grains above this size threshold regarded as tree birch (cf. Mäkelä and Hyvärinen 1998).

Coprophilous fungal spores were identified using the notes and photographs in Van Geel et al. (2003), with the prefix HdV- (Hugo-de-Vries laboratory, University of Amsterdam) added to the spore ‘type’ numbers (Feeser and O’Connell 2010; Schofield and Edwards 2011). Any apparently unique non-pollen palynomorphs (NPPs) encountered in this study are prefixed UoA- (University of Aberdeen). Coprophilous fungal spores are expressed as a percentage of the TLP sum. Microscopic charcoal was quantified through areal measurement (Patterson et al. 1987; Conedera et al. 2009; Mooney and Tinner 2011). Only black, opaque, angular particles with a length ≥ 5 μm were considered (Clark 1988). Charcoal concentrations (cm^2^ cm^-3^) were calculated to obtain charcoal to pollen ratios (C:P). Calculations of rarefaction—a measure of estimated species richness—were made using *psimpoll (*Bennett 2020b).

Pollen data were collated using Tilia 2.0.b.4 software and diagrams were created in TGView 2.0.2 (Grimm 1990). The placement of local pollen assemblage zones (LPAZs) was assisted through cluster analysis of the terrestrial pollen taxa using CONISS (Grimm 1987, 1990). Influx diagrams (palynomorphs cm^-2^ year^-1^) were employed to provide absolute and independent measures of pollen abundance for selected taxa (Davis and Deevey 1964).

### AMS ^14^C dating

Peat samples were disaggregated overnight by immersion in 10% NaOH, sieved through a nest of 250, 180 and 120 μm meshes and residues were inspected using a Nikon SMZ645 stereoscopic zoom microscope (x8-50 magnification). Selected plant macrofossils were removed from the sample residues and stored in distilled H_2_O and a drop of 10% HCl. Where suitable terrestrial macrofossils were unavailable, the humic acid fraction of 1 cm^3^ (bulk) peat samples was used. Samples were dated at the Scottish Universities Environmental Research Centre (SUERC), East Kilbride. Radiocarbon dates were calibrated using CALIB Version 7.0html (Stuiver and Reimer 1993) and the IntCal13 calibration curve (Reimer et al. 2013), and they are reported at the 2σ confidence level.

### Age-depth models

Age-depth models were produced using both ‘classical’ (*Clam*; Blaauw 2010) and Bayesian (*Bacon*; Blaauw and Christen 2011) software. Various model settings within *Clam* were explored (each run with 10,000 iterations) and those with the best ‘goodness of fit’ (GOF) were selected. In *Bacon*, models were run (> 6.5 million iterations) with different combinations of prior settings for deposition rate and accumulation shape. Priors were set so that the curve intersected the bulk of the probability distributions of the calibrated radiocarbon age ranges, using deposition rates that are considered reasonable for mires (cf. Mauquoy et al. 2002; Goring et al. 2012). Exact details of model settings are provided in the figure captions.

## Results

### Gammelhemmet

#### Lithostratigraphy

The stratigraphy comprises peat (79 cm in depth) resting upon a base of sand and gravel-rich grey clay that is probably of glacial origin. Fine herbaceous rootlets and woody detritus are visible throughout the peat. Further details of the pollen-analysed sequence (13-56 cm), together with Troels-Smith formulae for the deposit, are provided in Table 1.

**Table 1:** Lithostratigraphy of the GAM sequence described using Troels-Smith (1955) formulae and written descriptions. Only the section above 56 cm was pollen-analysed

Depth (cm)	Troels-Smith formula	Unit description
21–13	Sh3 Th^2^1 As+ Dl+ Nigr 4 Strat 0 Sicc 2+ Elas 0 Lim n/a	Well-humified black peat containing herbaceous rootlets and traces of clay and woody fragments.
64–21	Th^2^2 As1 Sh1 Dl+ Nigr 3+ Strat 0 Sicc 2+ Elas 0 Lim 0	Moderately-humified dark brown clay-rich peat with abundant herbaceous rootlets and traces of herbaceous stems and woody detritus.

#### Chronology

**Fig. 3:**
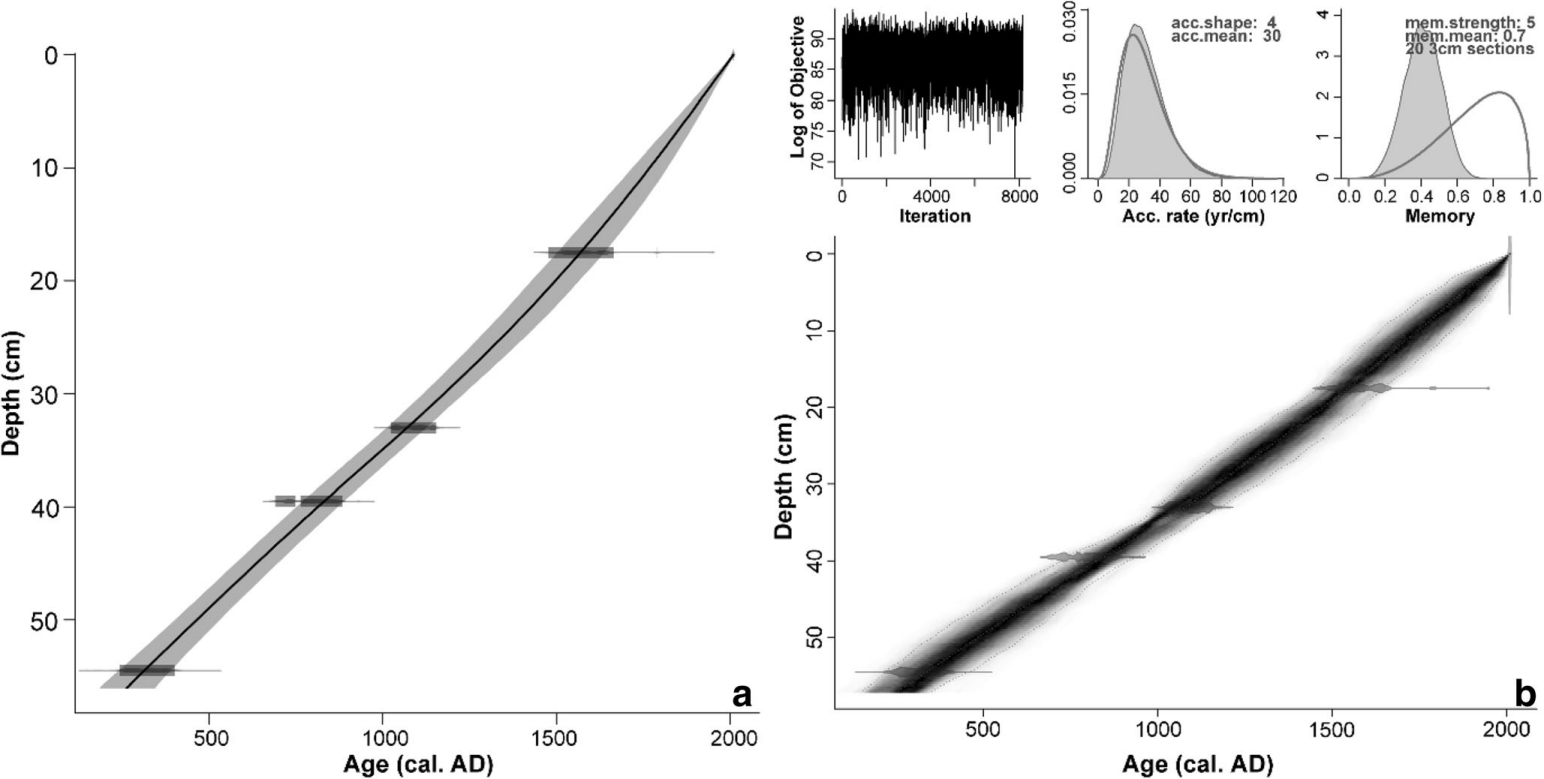
Age-depth models for Gammelhemmet (GAM) produced using (**a**) *Clam* (Blaauw 2010) and (**b**) *Bacon* (Blaauw and Christen 2011). Both models consider all radiocarbon measurements on *Betula* twigs/bark and humic acid (Table 2). With *Clam*, the best goodness of fit (GOF = 5.62) was achieved with a smooth spline, whereas in *Bacon* optimal results were achieved using a prior deposition rate (acc.mean) of 30 year cm^-1^ and an accumulation shape (acc.shape) of 4, a section thickness of 3 cm, a memory strength (mem.strength) of 5, and a memory mean (mem.mean) of 0.7

Radiocarbon dates are presented in Table 2. *Clam* and *Bacon* age-depth models resulted in near-identical chronologies (Fig. 3). Preference was given to the latter (more conservative) model. This age-depth model differs from that of Khorasani et al. (2015) in that no hiatus at the top of the sequence has been inferred here or seems to be indicated; deposition times are roughly linear throughout the sequence at ~ 30 year cm^-1^.

**Table 2:** Radiocarbon dates and ± 2σ calibrated age ranges for the GAM sequence

Depth (cm)	Lab code	Material	^14^C year BP (± 1σ)	Cal. year BC/AD (± 2σ)	δ^13^C (‰)
18–17	SUERC-36609	Peat (humic acid)	295 ± 40	AD 1477–1791	− 29.5
33	SUERC-27813	*Betula* bark	950 ± 30	AD 1024–1156	− 29.6
40–39	SUERC-38123	*Betula* twig	1225 ± 30	AD 690–885	− 28.1
54.5	SUERC-27814	*Betula* bark	1720 ± 35	AD 242–403	− 30.4

#### Palynology

**Fig. 4:**
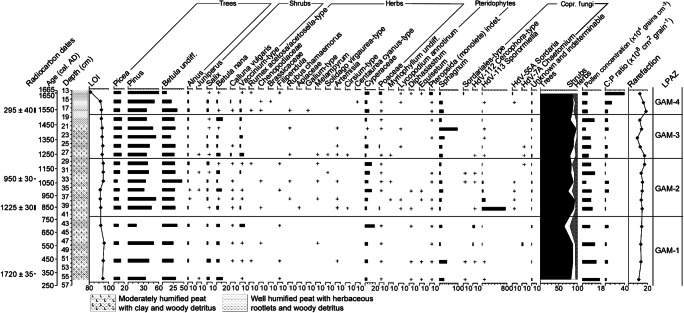
Percentage pollen diagram for Gammelhemmet (GAM) showing trees, shrubs and heaths, and herbs (sum ≥ 500 TLP) as well as aquatics, pteridophytes and coprophilous fungal spores. Also included are the calibrated and uncalibrated 14C ages, the lithological column for the sequence, the loss on ignition (LOI) values, microscopic charcoal expressed as charcoal to pollen (C:P) ratio and the rarefaction index. Rare types (< 1%) are indicated by a + symbol

**Fig. 5:**
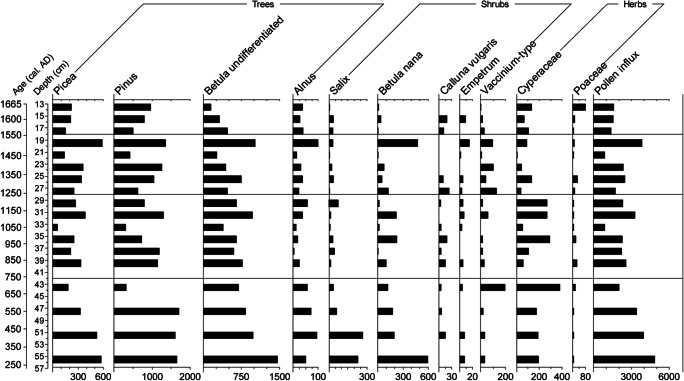
Pollen influx measured in grains cm^-2^ year^-1^ for selected trees, shrubs and herbs at Gammelhemmet (GAM). Note the differences in scaling of the x-axes

The pollen spectra (Figs. 4 and 5) seem to retain their stratigraphic integrity and display reasonably sharp and intelligible changes, supporting the absence of a hiatus in the sequence (at least below 13 cm depth). Four LPAZs can be distinguished in the percentage pollen diagram (Fig. 4). Their key features, including the patterns recorded in the influx diagram (Fig. 5), are summarized in Table 3.

**Table 3:** Description of the LPAZs at Gammelhemmet (GAM)

LPAZ	Depth (cm)	Cal. age range (AD)	Diagnostic pollen and spore characteristics
GAM-4	18–13	1545–1665	*Betula* undiff. declines (35–10%), *Pinus* increases (35–60%), herbs include traces of *Rumex*-type, Chenopodiaceae, Brassicaceae, *Centaurea cyanus* and coprophilous fungal spores of HdV-113 complemented by HdV-55A, especially at 14-13 cm, when LOI falls to 82% and C:P increases.
GAM-3	28–18	1230–1545	*Pinus* dominates (40–60%), *Vaccinium*-type consistently > 1%, most herbaceous taxa fade out, Cyperaceae reduced. *Sphagnum* peaks, coprophilous fungal spores reduced (diversity and abundance).
GAM-2	42-28	750–1230	*Pinus* and *Betula* dominate, the non-arboreal fraction includes traces of *Artemisia*, Asteraceae, Chenopodiaceae, *Juniperus*, *Plantago*, Poaceae and increasing levels of Cyperaceae. *Sphagnum* reduced, greater diversity and abundance of coprophilous fungal spores (40-31 cm) and dip in LOI (91%; 42–35 cm), C:P low.
GAM-1	56-42	290–750	*Pinus* dominates (up to 50%), *Vaccinium*-type 10% at 44–43 cm, herbs include traces of *Rumex*-type, Chenopodiaceae, *Melampyrum*, *Artemisia* and increasing levels of Cyperaceae. Coprophilous fungal spores present, C:P and LOI low.

### Hornmyr

#### Lithostratigraphy

The stratigraphy at Hormmyr comprises of a poorly-humified and fibrous peat extending to a depth of at least 95 cm. The full depth of the peat could not be established due to the presence of wood in the profile. Woody detritus is apparent below 29 cm, and this becomes increasingly abundant towards the base of the sequence. The deposit is further described in Table 4.

**Table 4:** Lithostratigraphy of the HORN sequence described using Troels-Smith (1955) formulae and written descriptions

Depth (cm)	Troels-Smith formula	Unit description
11–9	Sh4 As+ Dh+ Nigr 3 Strat 0 Sicc 2+ Elas + Lim 0	Well-humified dark brown peat with traces of clay and herbaceous rootlets.
29–11	Th^1^4 TSphag^0^+ Nigr 2 Strat 0 Sicc 2 Elas 0 Lim 0	Poorly-humified dark yellow-brown fibrous peat composed of herbaceous stems and rootlets.
70–29	Th^0^4 Dl+ Nigr 2 Strat 0 Sicc 2 Elas 0 Lim 0	Poorly-humified light yellow-brown peat composed of herbaceous stems and rootlets. Woody stems and rootlets occur below 55 cm.
87–70	Th^1^4 Dl++ Sh+ Nigr 2 Strat 0 Sicc 2 Elas + Lim 0	Poorly-humified dark yellow-brown fibrous peat containing herbaceous stems and rootlets as well as woody stems and rootlets (becoming more abundant below 74 cm).
95–87	Th^2^2 Sh2 Dl++ Tl^1^+ Nigr 4 Strat 0 Sicc 2 Elas 1 Lim 0	Black moderately humified peat composed of herbaceous and woody stems and rootlets.

#### Chronology

**Fig. 6:**
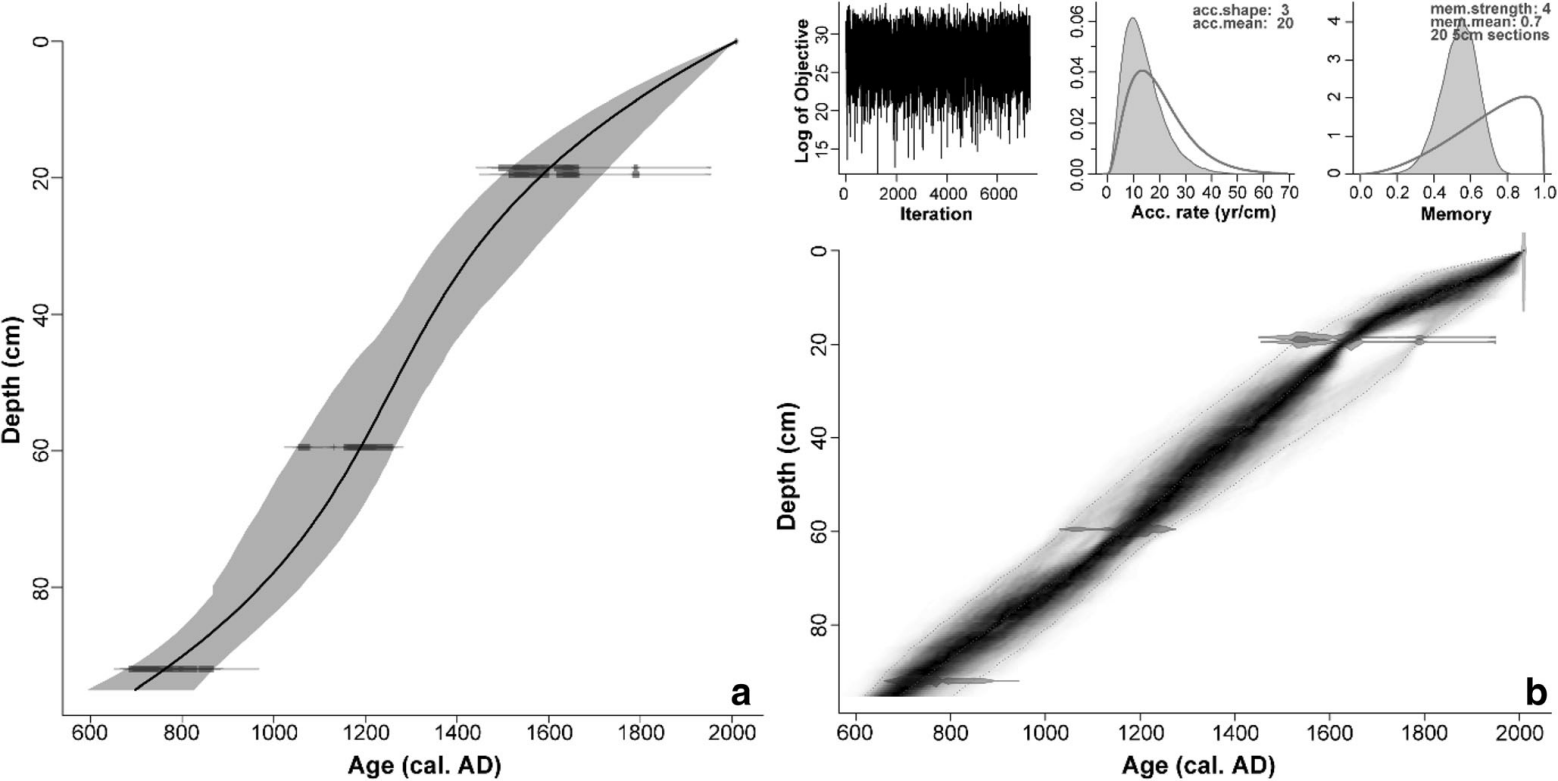
Age-depth models for Hornmyr (HORN) produced using (**a**) *Clam* (Blaauw 2010) and (**b**) *Bacon* (Blaauw and Christen 2011). Both models consider all radiocarbon measurements on *Betula* twigs, *Carex* nutlets and utricles and humic acid (Table 5). With *Clam*, the best goodness of fit (GOF = 1.49) was achieved with a polynomial regression of order 3 (smooth), whereas in *Bacon*, optimal results were achieved using a prior deposition rate (acc.mean) of 20 yr cm^-1^ and an accumulation shape (acc.shape) of 3, a section thickness of 5 cm, a memory strength (mem.strength) of 4 and a memory mean (mem.mean) of 0.7

Radiocarbon dates for HORN are presented in Table 5. Age-depth modelling with *Clam* and *Bacon* produced similar results and the *Bacon* model (Fig. 6) has been adopted.

**Table 5:** Radiocarbon dates and ± 2σ calibrated age ranges for the HORN sequence

Depth (cm)	Lab code	Material	^14^C year BP (± 1σ)	Cal. year BC/AD (± 2σ)	δ^13^C (‰)
19–18	SUERC-36608	*Carex* nutlets and utricles	285 ± 35	AD 1490-1795	− 23.2
20–19	SUERC-27818	Peat (humic acid)	275 ± 30	AD 1499-1795	− 27.3
59.5	SUERC-27819	*Betula* twig	850 ± 30	AD 1052-1261	− 27.0
92	SUERC-27820	*Betula* twig	1245 ± 30	AD 682-870	− 29.3

#### Palynology

**Fig. 7:**
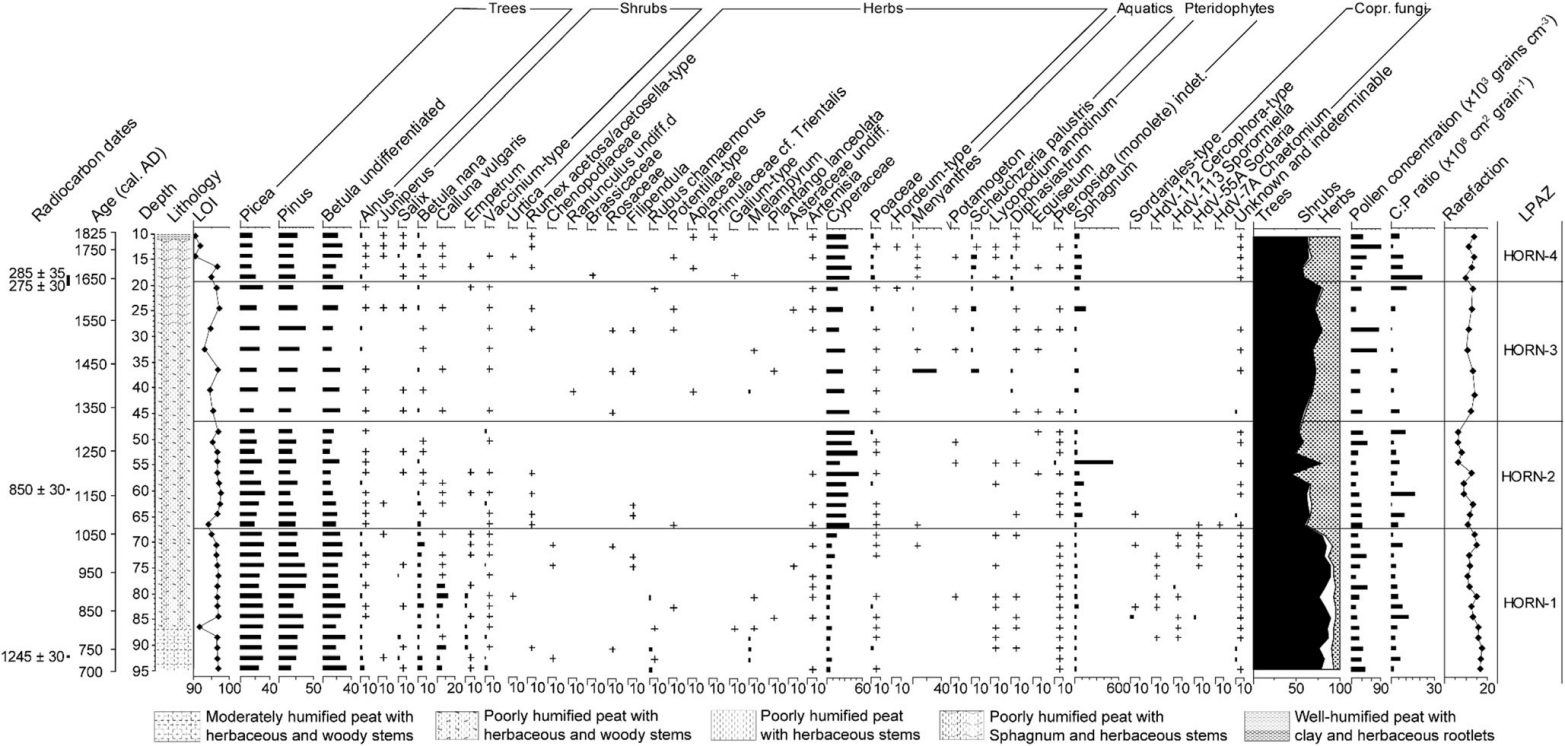
Percentage pollen diagram for Hornmyr (HORN) showing trees, shrubs and heaths and herbs (sum ≥ 500 TLP), aquatics, pteridophytes and coprophilous fungal spores. Also included are the calibrated and uncalibrated 14C ages, the lithological column for the sequence, the loss on ignition (LOI) values, the summary diagram, microscopic charcoal expressed as charcoal to pollen (C:P) ratio and the rarefaction index. Rare types (< 1%) are indicated by a + symbol

**Fig. 8:**
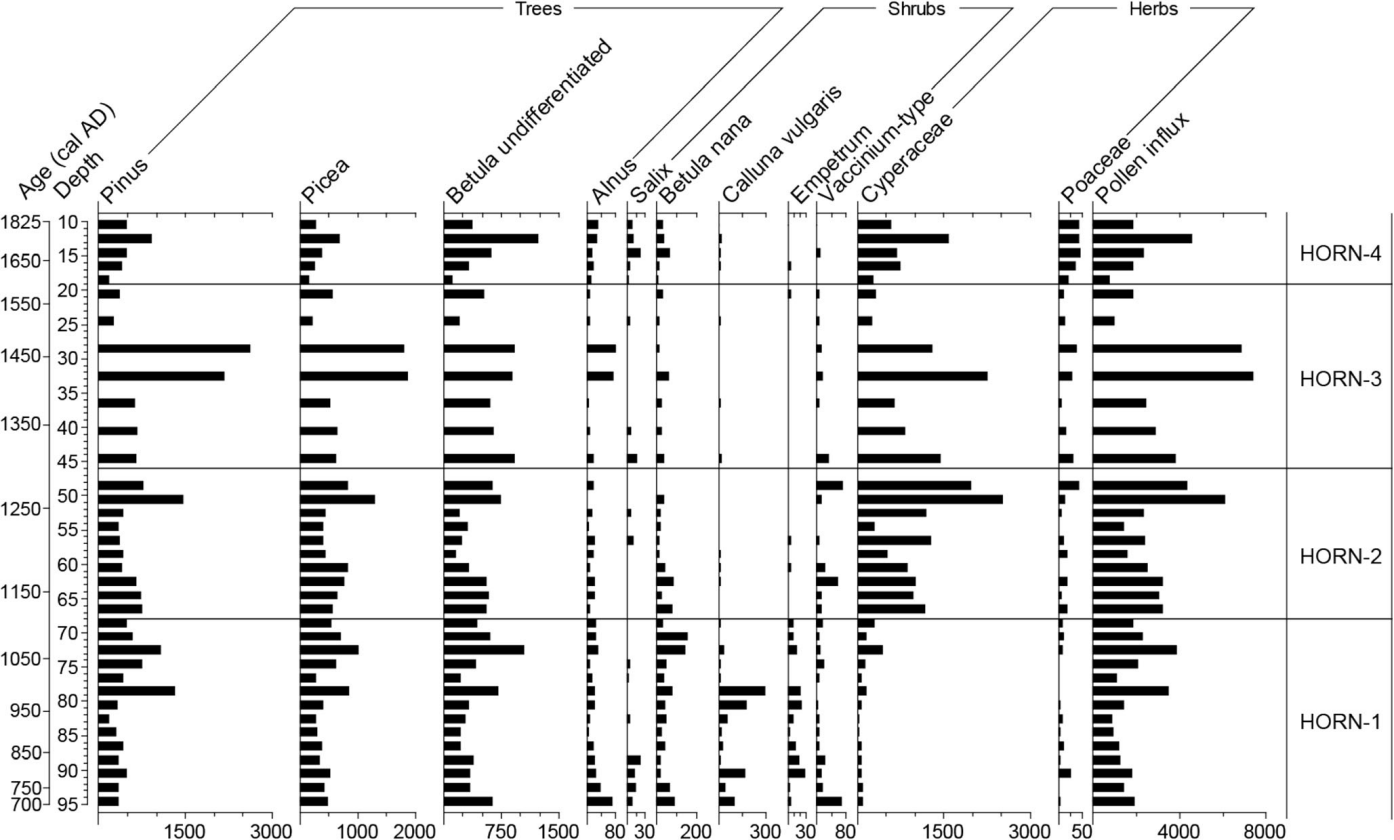
Pollen influx measured in grains cm^-2^ year^-1^ for selected trees, shrubs and herbs at Hornmyr (HORN). Note the differences in scaling of the x-axes

The palynological sequence (Figs. 7 and 8) appears to be undisturbed and is characterised by relatively pronounced and clearly defined changes. Four LPAZs can be distinguished in the percentage pollen diagram (Fig. 7). Their key features, including the patterns recorded in the influx diagram (Fig. 8), are summarized in Table 6.

**Table 6:** Description of the LPAZs at Hornmyr (HORN)

LPAZ	Depth (cm)	Cal. age range (AD)	Diagnostic pollen and spore characteristics
HORN-4	19–10	1655–1815	*Pinus* and *Picea* reduced, traces of *Urtica*, *Rumex*-type, Brassicaceae, *Potentilla*- type, Apiaceae, *Galium*-type and *Artemisia*. *Scheuchzeria* remains high, *Menyanthes* present. No coprophilous fungal spores, C:P high, LOI low.
HORN-3	46–19	1330–1655	Traces of *Rumex*-type, *Ranunculus* undiff. Rosaceae/*Filipendula*, Apiaceae, *Melampyrum*, *Plantago lanceolata* and *Artemisia.* Aquatics present in the top half of the LPAZ (traces of *Menyanthes*, *Scheuchzeria* 1–5%), C:P low, LOI fluctuates.
HORN-2	68–46	1055–1330	Traces of *Rumex*-type, *Filipendula*, *Potentilla* and *Artemisia* at the onset; Cyperaceae and *Sphagnum* increase. Coprophilous fungal spores absent, C:P fluctuates, rarefaction decreases, LOI high.
HORN-1	95–68	695–1055	*Picea*, *Pinus* and *Betula* undiff. dominate, *Betula nana* and *Calluna* abundant. *Salix*, *Melampyrum* and *Rubus chamaemorus* >1% but reduced to traces as coprophilous fungal spores appear (89-88 cm). Traces of *Juniperus*, *Urtica*, *Rumex*-type, Chenopodiaceae, *Plantago lanceolata*, *Achillea*-type (Asteraceae) and *Artemisia*. LOI high except for dip at 87–86 cm.

## Palaeoecological interpretation

### Gammelhemmet

#### LPAZ GAM-1: ~ AD 290–750 (56–42 cm)

The timing of this LPAZ broadly coincides with the cold and wet Pre-Medieval Cold Period (~ AD 300–900; Cowling et al. 2001; St. Amour et al. 2010), which might explain the dominance of *Pinus* over *Betula* and low C:P through the suppression of natural forest fire activity. Wetter substrates would be favourable to many species within the Cyperaceae as well as *Vaccinium*-type (e.g. *V. oxycoccos* and *V. microcarpum*, both abundant in northern Sweden; Mossberg and Stenberg 2010), which is seen to increase at the top of the zone (visible in both percentage and pollen accumulation rate [PAR] diagrams—albeit in the single uppermost spectrum in the percentage profile). Conditions may have become increasingly unfavourable for *Pinus*; this is supported by entomological evidence, which indicates wet conditions coinciding with an increasingly open landscape from the Early Iron Age into the Late Iron Age (Khorasani et al. 2015). The presence of coprophilous fungal spores of *Sporormiella*-type (HdV-113) and *Cercophora*-type (HdV-112) at low frequencies suggests that the site was visited by animals.

#### LPAZ GAM-2: ~ AD 750–1230 (42–28 cm)

This zone largely coincides with the interval sometimes ascribed to the warmer and drier climate of the so-called Medieval Warm Period (~ AD 900–1200; Briffa et al. 1992), but there are no strong changes in the palynological signal. There is a reduction in *Sphagnum* spore representation in the upper portion of the LPAZ, and Cyperaceae is consistently in evidence. An increase in the abundance of, or regularity with which animals visited the site, could explain the heightened frequencies and greater variety of coprophilous fungal spores from ~ AD 845–1125. Similar increases in coprophilous fungal spores at an ombrotrophic peat bog in high latitude northern Norway, particularly of *Sporormiella* and *Sordaria*, have been linked to reindeer grazing (Balascio et al. 2020). The more continuous presence of pollen of ruderal apophytic taxa (e.g. *Artemisia*, Asteraceae, Chenopodiaceae and *Solidago*-type; Behre 1988; Aronsson 1994; Josefsson et al. 2009) and those regarded as responding favourably to forest grazing (e.g. *Melampyrum* and *Juniperus*; Behre 1981; Poska et al. 2004) may support this suggestion. A combination of such taxa was attributed to general disturbance, forest grazing and increased nutrient availability at reindeer herding sites in northern Sweden (Aronsson 1991, 1994). A slight increase in the minerogenic input could be explained by heightened levels of erosion due to trampling of dry land areas surrounding the mire. Values for Cyperaceae are relatively lower in samples where the abundance of *Sporormiella-*type is greatest, but recover when the suggested grazing pressure on the bog is reduced—a trend that is most obvious in the PAR diagram; *Salix* is also reduced to trace values, but soon recovers to pre-LPAZ GAM-2 levels. It is impossible to say with certainty that reindeer were responsible, but such an explanation seems plausible given their preference for feeding on bogs where *Salix* spp. and Cyperaceae (*Eriophorum* spp. and *Carex* spp. especially) are available in abundance (Renbeteslära 1982; Flenniken 2007; Yu et al. 2011). The signal is unlikely to be related to moose grazing as, when feeding, they target the upper (30–300 cm) parts of shrubs and trees (e.g. *Pinus sylvestris, Salix* spp.*, Juniperus communis, Sorbus aucaparia, Populus tremula, Alnus incana* and *Betula pendula*) in summer and winter; these are more easily accessible than forbs and herbs given the disparity in height between plant and animal (Shipley et al. 1998; Wam and Hjeljord 2010). Low and stable C:P, similar to LPAZ GAM-1, indicates low intensity or infrequently occurring forest fires; the slightly increased levels of charcoal at ~ AD 1000–1050 may reflect a contribution from domestic fires given the onset of recurrent Sami presence in the parish between AD 1000 and 1350 (Zachrisson 1984). This LPAZ may correspond to the period when the development of a woodland mosaic with increasingly abundant open patches was advanced by Khorasani et al. (2015), possibly driven by Sami activity across the wider area.

#### LPAZ GAM-3: ~ AD 1230–1545 (28–18 cm)

This zone coincides with the first half of the colder and wetter Little Ice Age (LIA, ~ AD 1200–1850; Cowling et al. 2001; Matthews and Briffa 2005). Cyperaceae is reduced in percentage as well as PAR terms, despite a preference for wetter conditions among many of this family’s constituent species. In the high Arctic, however, *Carex aquatilis stans*, *C. membranacea*, and *Eriophorum angustifolium* (the former two being widespread in northern Sweden; Mossberg and Stenberg 2010) have been shown to respond in unison with changes in temperature (Hill and Henry 2011). Lowered temperatures and greater wetness would also favour *Sphagnum*. Although an absence of spores does not necessarily have to relate to an absence of herbivore activity (Perrotti and van Asperen 2019; Van Asperen et al. 2019), the reduction of *Sporomiella*-type spores (HdV-113), the disappearance of *Cercophora*-type (HdV-112) and *Sordaria*-type (HdV-55A), and a strongly reduced presence of pollen of ruderals and taxa related to general landscape openness (e.g. *Artemisia*, *Solidago*-type, *Cirsium*-type and Poaceae) beyond the basal sample in the zone, suggest a reduction in the intensity of grazing at the site and/or lower numbers of herbivores. This is supported by a decrease in species richness as indicated by the rarefaction curve.

#### LPAZ GAM-4: ~ AD 1545–1665 (18–13 cm)

The drop in LOI at 14-13 cm (~ AD 1640–1665) coincides with a steep rise in C:P and a decline in *Betula,* while *Pinus* increases through the zone. A concomitant decline in *Picea* is more evident in the PAR diagram. Considering that the envelope of uncertainty on the age-depth model extends to AD 1755, it is possible that these changes relate to slash-and-burn clearance as practised by the ‘Forest Finns’, who first settled in this region (in Örträsk) in AD 1678. *Pinus* may have benefitted from an increased frequency or intensity of fires; it is resistant to burning because of its thick and insulating bark, it is able to recover quickly and to resist fungal and insect attacks following fire damage, and its roots are protected as they penetrate relatively deeply into the soil (Zackrisson 1977).

Trace amounts of Brassicaceae, *Centaurea cyanus*, Chenopodiaceae, and *Rumex*-type pollen suggest disturbance, possibly through arable farming and/or settlement (Bellanger et al. 2012). Given the surface area of the mire (~ 1.5 ha), pollen recruitment is likely to derive from extra-local or regional pollen source areas, but the limited dispersal of pollen beyond the boundaries of cultivated fields situated within boreal forests (~ 20–30 m, e.g. Vuorela 1973; Hall 1988) might suggest that farming was taking place in close proximity to the bog. Low levels of agricultural indicator taxa could also be explained if high-pollen producers such as *Pinus* and *Betula* are masking the signal (cf. Hicks 1985). In addition, any arable farming probably occurred on a relatively small scale as it was of minimal importance in these areas compared to stock raising until well into the nineteenth century (Engelmark 1976; Gadd 2011). Around AD 1700, only ~ 2% of Sweden’s land area was under arable production, most of which was not in Norrland (Gadd 2011). The absence of cereal-type pollen may support the suggestion that clearance was not aimed at creating arable land, although the pollen of Cerealia are not well dispersed (Vuorela 1973).

An increase in Cyperaceae pollen percentages and accumulation rates in GAM-4 may represent a local response to the ~ AD 1550–1760 wet shift as recorded by Van der Linden et al. (2008). Alternatively, its coincidence with possible slash-and-burn and the increase in Poaceae at 14–13 cm suggests that changes in the representation of sedges may relate to the creation of pasture land and/or hay making on both wet and dry meadows (Segerström and Emanuelsson 2002; Josefsson et al. 2009). The reoccurrence of spores of Sordariales-type and *Sordaria* (HdV-55A), albeit slight, is noteworthy as the presence of coprophilous fungal spores during meadow phases has been linked to both grazing and the manuring of hay meadows (Graf and Chmura 2006). Spores of Sordariales-type, however, include non-obligate coprophilous taxa (Lundqvist 1972), and in the absence of a (significant) response in *Sporormiella*-type—the most reliable indicator of herbivore presence—it cannot be concluded that manuring of, or grazing on, the bog occurred.

Khorasani et al. (2015) recorded palaeoentomological evidence that suggested the use of fire for clearance and possible drainage for agricultural purposes in their S3 sample (20–30 cm depth), which they dated to AD 1446–1633. We suggest that the activities recorded by them pertain to the seventeenth century agricultural settlement as recorded in the topmost samples of the palynological record.

### Hornmyr

#### LPAZ HORN-1: ~ AD 695–1055 (95–68 cm)

From ~ AD 755, the striking presence of coprophilous fungal spores suggests animal grazing on the mire, possibly by reindeer whose preference for *Salix* as a food source would explain its decline in the pollen percentage and influx records. Pollen of *Juniperus*, *Urtica*, *Rumex acetosa*, Chenopodiaceae, *Melampyrum*, *Plantago lanceolata*, Asteraceae and *Artemisia*, which collectively occur throughout this LPAZ, suggest a combination of forest grazing, general disturbance and increased nutrient availability. *Rumex acetosa/acetosella is* considered of particular importance as an anthropogenic indicator in northern Fennoscandian forest settings by Josefsson et al. (2009). There is an absence of a rise in Poaceae that generally accompanies this suite of pollen types, suggesting that grazing may be inhibiting the flowering of grasses. The dip in LOI at ~ AD 810, admittedly a single level, resembles that recorded at a reindeer herding pen (*renvall*) at Akkajärvi, northern Sweden, which was linked to the creation of a small clearance in the forest for an extension to the *renvall* (cf. Freschet et al. 2014; Kamerling et al. 2017). At the winter village of Einehlammet in eastern Finnish Lapland, Hicks (1993) found a decline in LOI coincided with a minor peak in C:P, which was interpreted as belonging to small, local fires. A short-lived increase in C:P is recorded in HORN-1 from around AD 855, which may relate to smudge fires (lingering smoke-producing fires used to protect reindeer from mosquitoes [cf. Garriott 1899; Aronsson 1991]), and/or domestic fires set by the Sami within the vicinity of the bog, and/or possibly fires set by Sami to promote and sustain reindeer lichen-dominated ground vegetation for winter grazing (Hörnberg et al. 2018). C:P is generally lower towards the top of the zone, which may reflect less intensive use of the area, although fluctuations in fire incidence may arise from natural forest fires.

During periods of disturbance, rarefaction values might be expected to increase (Birks and Line 1992; Gaillard 2007) as was found to be the case in reindeer grazing areas in the tundra-heath of northern Norway (Olofsson et al. 2001) and at a herding pen (*renvall*) at Akkajärvi (Kamerling et al. 2017). However, the impacts of reindeer on the vegetation may vary in different climatic, geographical and biotic contexts (Bernes et al. 2013) and whether species richness will respond positively or negatively to reindeer grazing depends on its intensity and the environmental heterogeneity (Pajunen et al. 2008). If the coprophilous fungal spore signal at Hormmyr relates to Sami reindeer herding, it largely pre-dates the period (~ AD 1000–1350) of archaeologically well-documented Sami activity in Lycksele parish (Zachrisson 1984).

#### LPAZ HORN-2: ~ AD 1055–1330 (68–46 cm)

The disappearance of coprophilous fungal spores suggests that grazing at the site had largely ceased at this time. The preferred food source of reindeer, Cyperaceae, shows markedly increased pollen representation relative to the previous LPAZ. As noted earlier, some of the species within this family that have a common distribution across northern Sweden respond positively to climate warming. In the absence of grazing pressure, it is possible that certain species of Cyperaceae (e.g. *Carex aquatilis stans* and *C. membranacea*) were able to respond positively to the ameliorating conditions of the Medieval Warm Period (cf. Hill and Henry 2011). An end to grazing—which may have included feeding on mosses when access to young shoots of *Salix*, *Eriophorum* spp. and *Carex* spp. was limited (Yu et al. 2011)—might also explain the increased abundance of *Sphagnum*. A caveat is that in forest-tundra ecotones in northwestern Finnish Lapland, bryophytes have been shown to respond negatively to a lack of reindeer grazing (Pajunen et al. 2008). The increase in *Sphagnum* could also be related to (local) changes in surface wetness. C:P levels remain similar to those witnessed during HORN-1, and in the absence of other indicators of human impact, the microscopic charcoal is probably attributable to natural forest fires.

#### LPAZ HORN-3: ~ AD 1330–1655 (46–19 cm)

Colder summers during the LIA may be responsible for the overall reduction in Cyperaceae throughout this zone (most clearly visible in the PAR diagram), while the rise in the abundance of aquatic taxa (*Menyanthes*, *Potamogeton* and *Scheuchzeria palustris*) from ~ AD 1435 may signify an increase in shallow pools on the bog. Colder and wetter summers would further explain the reduced frequency of natural forest fires, as expressed by the decline in C:P, thus allowing an overall increase in tree taxa, including *Alnus* spp., which has a preference for wetter substrates. The combination of traces of ruderals (*Rumex*-type, *Plantago lanceolata*, *Artemisia*), and indicators of meadows (*Ranunculus* undiff., *Filipendula*), general open land (Apiaceae) and forest grazing (*Melampyrum*), suggest disturbance and an opening of the landscape. This signal does not appear to relate to an early onset of Finnish agricultural impact as there is little palynological evidence for slash-and-burn cultivation (C:P is low and there are no obvious reductions in percentages or PARs of arboreal pollen types). If impacts arising from farming are discounted, it is possible that Sami activity within the vicinity of the bog may have produced this signal.

#### LPAZ HORN-4: ~ AD 1655–1815 (19–10 cm)

This LPAZ appears to represent Finnish colonization of the region, with swiddening expressed as a reduction in *Picea* and a rise in C:P. *Picea*-dominated forest, with its moist soils, was often targeted for clearance as it offered the most suitable substrate for cultivation (Engelmark 1976; Zackrisson 1976; Hicks 1985). The palynological signal recorded here, given the moderately large size of the mire, probably represents clearance activity that is beyond the immediate bounds of the mire. Possible evidence for cultivation and an opening of the landscape are expressed as greater community diversity, and the appearance and/or increase of taxa relating to arable farming and open, grazed and trampled landscapes with enhanced soil nutrient levels (e.g. *Hordeum*-type, Brassicaceae, *Urtica*, *Rumex acetosella*, *R. acetosa*, Apiaceae and *Artemisia*). *Hordeum*-type is a category that includes the pollen of wild grasses such as *Elytrigia repens* and *Glyceria fluitans* (Andersen 1979). The latter is rarely found in interior northern Sweden, but the former is very common and occurs mainly on cultivated soils (Mossberg and Stenberg 2010). Given the limited capacity for dispersal of pollen of cultivated plants beyond the boundaries of cultivated fields set within boreal forests (similar to Gammelhemmet), cultivation may have occurred within close proximity (~ 20–30 m) of the sampling location. The timing of these activities fits the known settlement history of the wider Lycksele area, although if the chronological estimates based on the age-depth model are accepted, farming at Hornmyr appears to pre-date the documented establishment age of the village (AD 1766).

The increase in percentages and PARs of Cyperaceae and Poaceae suggests that the site may have been used as a (wet) hay meadow (cf. Segerström and Emanuelsson 2002). The presence of *Menyanthes* and *Scheuchzeria* through this LPAZ intimates that pools with standing water were maintained, meaning that the posited hay meadow would not necessarily need to have been purposefully flooded to improve its productivity (cf. Elveland 1979; Vasari and Väänänen 1986; Vasari 1988). The absence of coprophilous fungal spores could indicate that the mire was not manured or used as pasture.

## Discussion

### Reindeer grazing in the Lycksele region ~ AD 800–1100

At both Gammelhemmet and Hornmyr, various types of coprophilous fungal spores (HdV-7a, 55a, 112 and 113) are registered between ~ AD 845–1125 (part of GAM-2) and ~ AD 780–1055 (part of HORN-1) respectively. The similar timing of these patterns at both sites suggests a common cause, and although it may not be possible to directly relate spore abundance to animal abundance/biomass (Davies 2019), the presence of such spores most likely indicates an increased abundance of grazing herbivores in the Lycksele region. Considering the limited dispersal capacity of coprophilous fungal spores from their fruiting bodies (from ≤ 2.5 m to a maximum of tens of meters; Ingold 1971; Yafetto et al. 2008; Davies 2019), herbivores must have been regularly present in numbers along the margin of the bogs, and probably also on their surfaces. The spore assemblages are dominated by *Sporormiella*-type (HdV-113), which is considered to be the most useful indicator of herbivore presence (Davis and Shafer 2006; Raper and Bush 2009; Feranec et al. 2011). Different species within this genus grow on the dung of large birds such as grouse (Richardson 1972, 2001; Wood et al. 2011), domestic cattle (West 2003), lagomorphs, deer, horses and porcupines (Ahmed and Cain 1972). It is impossible to distinguish morphologically between fungal spores produced by different species within the genus *Sporomiella* (Miller and Huhndorf 2005) and therefore no inferences can be made as to the type of animal that produced the dung from which they are likely to have originated. The impacts on the preferred food sources of reindeer, i.e. the negative response of *Salix* at Gammelhemmet during the phase of increased coprophilous spore abundance, and the recovery of Cyperaceae at Hornmyr at the time these spores disappeared, support the idea that reindeer used the bogs as pasture.

Reindeer browsing is extensive rather than of high grazing intensity, and it has been suggested that this would lead to minimal impact on the vegetation (Hicks 1985; Aronsson 1991), though Josefsson et al. (2009, 2010) argue that long-term reindeer herding over an extensive area would result in distinctive differences in forest structure and composition compared to surrounding areas. The suite of shrub and herb taxa recorded at both Gammelhemmet and Hornmyr during the postulated grazing phases (*Rumex*-type, Chenopodiaceae, *Melampyrum*, Asteraceae, *Artemisia*, and Poaceae at GAM-2; *Juniperus*, *Urtica*, *Rumex acetosa*, Chenopodiaceae, *Melampyrum*, *Plantago lanceolata*, Asteraceae and *Artemisia* at HORN-1; ~ AD 695–1055) includes the majority of taxa that were identified by Aronsson (1991) as related to reindeer gathering at *renvalls*, and listed by Behre (1981) as potential indicators of dry pasture and grazed forests. This suggests that (domesticated) reindeer were gathered within the vicinity of both bogs, or alternatively, that the number of wild reindeer was great enough across the wider Lycksele area, and their presence sufficiently regular, that it resulted in a region-wide impact on the vegetation. Unfortunately, little is known about wild reindeer migration patterns in northern Sweden, but by the time European settlers entered the region in the seventeenth century, it is reported that wild reindeer were abundant across the wider Lycksele area. The greatest concentration is noted as occurring in the area ~ 40–175 km to the south of Lycksele (Norstedt 2011).

The time interval under discussion here (~ AD 800–1100) largely pre-dates the ~ AD 1000–1350 period of archaeologically-documented Sami activity in the Lycksele parish area, even allowing for the inferred AD 1125 impact at Gammelhemmet. The apparent presence of wild reindeer may have been what attracted the Sami to this region as their subsistence was, at least in part, dependent upon reindeer hunting. Furthermore, the importance of access to furs increased during the Viking Age (~ AD 800–1050) when furs became an important trade commodity (Sawyer 2000). Whether the Sami simply followed the reindeer through the landscape as they hunted them, or whether they exercised some level of control over their movements and choice of grazing areas, is unclear. The use of smudge fires was one of the main methods of luring small and relatively tame herds of reindeer during the ‘true’ intensive reindeer herding period; this is considered to have begun during the seventeenth or eighteenth century (Aronsson 1991; Lundmark 2007) and was characterised by hunting and gathering while keeping small herds of relatively tame animals (Niklasson et al. 1994; Bergman et al. 2004; Müller-Wille et al. 2006). If intensive reindeer herding was already occurring across the wider Lycksele area during the ~ AD 800–1100 period, a rise in microscopic charcoal would be expected. At Hornmyr, a peak in C:P is recorded around ~ AD 855, although values of a similar magnitude can be seen during the following LPAZ when no other obvious indications of human impact are visible.

The apparent cessation of grazing within the Lycksele area is indicated by the decline in coprophilous fungal spores after ~ AD 1100. Sami activity in the wider area persisted for another 250 years after the cessation of animal presence at Hornmyr and was seemingly strongly reduced at Gammelhemmet. Although the pressure of hunting may have been detrimental to reindeer in the region, it is perhaps more likely that the Sami utilised some level of control over the pasturing grounds that the animals frequented.

### Separating contemporaneous signals for Sami activity and Nordic (agricultural) settlement

At both Gammelhemmet and Hornmyr, a clear signal for Finnish colonization supported by inferred slash-and-burn is seemingly evident in the palynological record, particularly through a positive response in microscopic charcoal. During the period of seventeenth century settlement, no obvious indication of Sami activity can be distinguished in the palynological record from Hornmyr. This may be a taphonomic artefact related to the limited dispersal capacity of pollen from areas of Sami reindeer herding activity within boreal forests (Hicks 1985; Aronsson 1991; Abramsson et al. 2006; Kamerling et al. 2017). Alternatively, it may simply mean that the Sami were not especially active within the vicinity of the mires. Contact between the Sami and Finns would have occurred at the winter market towns, although these groups need not have had much interaction beyond that. The increasing disturbance to reindeer grazing lands as colonization progressed, and the pressure of forced Christianisation (Broadbent 2010), may have caused the Sami to relocate, in a similar fashion to the Sami in Kuusamo, northern Finland during the second half of the seventeenth century (Hicks 1985). With just two spectra at Gammelhemmet covering the period of Finnish colonization, it is not possible to confidently state an absence of a palaeoecological signal for the Sami there.

If both cultures were active contemporaneously at Gammelhemmet and Hornmyr, the palynological signal produced by Finnish and Nordic settlers—reflecting livestock rearing through transhumance, supplemented by low-level cultivation—may be inseparable from any evidence for Sami reindeer herding activity, and indeed the Sami may have practised low-level cultivation of their own. Both activities are characterised by similar palynological indicators (these being increased levels of Poaceae, *Rumex*-type, *Plantago lanceolata*, *Artemisia*, Chenopodiaceae, *Solidago*-type, *Urtica*, *Juniperus*, and *Melampyrum* pollen, and the occurrence of coprophilous fungal spores). However, it should also be considered that the Sami would have visited the Lycksele *prästbord*, within which Gammelhemmet and Hornmyr are situated, only during the winter. The impact of Sami settlement and small herds on the vegetation under winter conditions is expected to have been limited (Aronsson 1991); Sami would have lived in fully mobile tents (*kåtor*; Manker 1968) and reindeer herds would have been relatively small after autumn slaughtering (Müller-Wille et al. 2006), with the animals largely feeding on lichens when vascular plants were unavailable (Renbeteslära 1982).

## Conclusions

By assessing a combination of palynological proxies, including microscopic charcoal and coprophilous fungal spores, this study shows that small-scale human impact can be identified in palynological sequences from boreal forest settings given the selection of sampling locations near to the postulated foci of activity. At both Gammelhemmet and Hornmyr, the palynological signals relating to grazing herbivores and arable agriculture are superimposed upon broader climate-controlled vegetational patterns. Archaeological evidence for the regional presence of herbivores is supported locally by the coprophilous fungal spore record (~ AD 845–1125 at Gammelhemmet and ~ AD 780–1055 at Hornmyr). Together with a reduction in the abundance of Cyperaceae, *Salix* and *Sphagnum* (the preferred food types of *Rangifer*), this suggests that reindeer may have grazed on these bogs. The timing of these events partly overlaps with an archaeologically documented period of Sami activity for the region dating to the late Iron Age and Early Middle Ages (~ AD 1000–1350). The Sami were perhaps drawn to the wider Lycksele area by the availability of reindeer for hunting and/or herding. The results demonstrate the importance of studies of coprophilous fungal spores in tracing the history of reindeer herding and grazing. They allow the establishment of the local (on-site) presence of herbivores where impacts on the vegetation from mobile hunter-herders are minimal and may not be otherwise distinguishable in the palynological record.

The impact of Finnish colonization in the parish is evident from ~ AD 1650. This is expressed in the form of high levels of microscopic charcoal, a reduction in the abundance of arboreal pollen, increased levels of Poaceae and/or Cyperaceae related to hay making on dry and wet meadows, and the establishment of ruderal plants associated with settlement, stock raising, and possibly cultivation. No contemporaneous signal for Sami and Finnish activity was discovered, either due to the limited dispersal of pollen of indicator taxa from locations of Sami interference, or because Sami activity did not coincide with that of Finnish agriculturalists around the sites investigated here.

## Data Availability

Data is available on request.
